# Anatomic stabilization techniques provide superior results in terms of functional outcome in patients suffering from chronic ankle instability compared to non-anatomic techniques

**DOI:** 10.1007/s00167-017-4730-4

**Published:** 2017-11-14

**Authors:** G. Vuurberg, H. Pereira, L. Blankevoort, C. N. van Dijk

**Affiliations:** 1 Department of Orthopaedic Surgery, Academic Medical Centre, Amsterdam Movement Sciences, University of Amsterdam, PO Box 22660, 1100 DD Amsterdam, The Netherlands; 2 Academic Center for Evidence Based Sports Medicine (ACES), Amsterdam, The Netherlands; 3 Amsterdam Collaboration for Health and Safety in Sports (ACHSS), Amsterdam, The Netherlands; 4 Centro Hospitalar Póvoa de Varzim – Vila do Conde, Póvoa de Varzim, Portugal; 5 ICVS/3B’s—PT Government Associated Laboratory, University of Minho, Braga, Guimarães, Portugal; 6 Ripoll y De Prado Sports Clinic: Murcia-Madrid—FIFA Medical Center of Excellence, Madrid, Spain

**Keywords:** Ankle instability, Chronic, Surgical treatment, Functional outcome

## Abstract

**Purpose:**

To determine the best surgical treatment for chronic ankle instability (CAI) a systematic review was performed to compare the functional outcomes between various surgical stabilization methods.

**Methods:**

A systematic search was performed from 1950 up to April 2016 using PubMed, EMBASE, Medline and the Cochrane Library. Inclusion criteria were a minimum age of 18 years, persistent lateral ankle instability, treatment by some form of surgical stabilization, described functional outcome measures. Exclusion criteria were case reports, (systematic) reviews, articles not published in English, description of only acute instability or only conservative treatment, medial ankle instability and concomitant injuries, deformities or previous surgical treatment for ankle instability. After inclusion, studies were critically appraised using the Modified Coleman Methodology Score.

**Results:**

The search resulted in a total of 19 articles, including 882 patients, which were included in this review. The Modified Coleman Methodology Score ranged from 30 to 73 points on a scale from 0 to 90 points. The AOFAS and Karlsson Score were the most commonly used patient-reported outcome measures to assess functional outcome after surgery. Anatomic repair showed the highest post-operative scores [AOFAS 93.8 (SD ± 2.7; *n* = 119); Karlsson 95.1 (SD ± 3.6, *n* = 121)], compared to anatomic reconstruction [AOFAS 90.2 (SD ± 10.9, *n* = 128); Karlsson 90.1 (SD ± 7.8, *n* = 35)] and tenodesis [AOFAS 86.5 (SD ± 12.0, *n* = 10); Karlsson 85.3 (SD ± 2.5, *n* = 39)]. Anatomic reconstruction showed the highest score increase after surgery (AOFAS 37.0 (SD ± 6.8, *n* = 128); Karlsson 51.6 (SD ± 5.5, *n* = 35) compared to anatomic repair [AOFAS 31.8 (SD ± 5.3, *n* = 119); Karlsson 40.9 (SD ± 2.9, *n* = 121)] and tenodesis [AOFAS 19.5 (SD ± 13.7, *n* = 10); Karlsson 29.4 (SD ± 6.3, *n* = 39)] (*p* < 0.005).

**Conclusion:**

Anatomic reconstruction and anatomic repair provide better functional outcome after surgical treatment of patients with CAI compared to tenodesis reconstruction. These results further discourage the use of tenodesis reconstruction and other non-anatomic surgical techniques. Future studies may be required to indicate potential value of tenodesis reconstruction when used as a salvage procedure. Not optimal, but the latter still provides an increase in functional outcome post-operatively. Anatomic reconstruction seems to give the best results, but may be more invasive than anatomic repair. This has to be kept in mind when choosing between reconstruction and repair in the treatment of CAI.

**Level of evidence:**

IV.

## Introduction

Despite a high incidence of lateral ankle ligament injuries [45], only a small proportion of the patients seeks medical care [2, 38, 42, 43, 46]. In instances where a patient has not responded favourably to conservative treatment (e.g. prolonged course of physiotherapy and/or bracing), surgical stabilization may be an appropriate option to restore function, depending on the patient’s needs and expectations [16, 33].

Tenodesis is the oldest surgical technique. It includes a non-anatomic reconstruction [16]. Currently, anatomic reconstruction or repair techniques are preferred in order to restore joint configuration and mechanics [1, 2, 14, 15, 33, 44]. The last technique that has been used is capsular shrinkage that uses local heat application to induce shrinkage of the anterior talofibular ligament (ATFL) and capsule [11, 29].

Many studies have shown the success of these techniques in treating CAI. Mabit et al. [31]. were the first to compare anatomic repair with non-anatomic reconstruction, showing superior short-term results (pain, symptoms, function) for anatomic repair. Other studies confirmed these results [18, 26–28]. Up till now only de Vries et al. [12]. published a Cochrane review on outcome and complications after different surgical stabilization techniques in patients with CAI. Despite comparisons of effectiveness between techniques, they concluded that there was insufficient evidence to support any surgical intervention over another. The previously published review has not focussed on the patient-reported outcomes after surgical ankle stabilization. Additionally, since then more research has become available.

If it is known which technique provides the best post-operative technical and functional outcome, then patient benefit and surgical results can be simultaneously optimized. For this reason, the objective of this systematic review is to determine the most effective surgical treatment in patients with CAI by providing a review of published studies and comparing functional outcomes after surgical stabilization.

## Materials and methods

### Search strategy

The research question of this review was: ‘what is the best surgical treatment strategy for patients with CAI based on patient-reported functional outcome?’ To answer this question a search was conducted in Pubmed, EMBASE, Medline and the Cochrane Library from 1950 up to April 2016, including the terms ‘surgical treatment’, ‘lateral’, ‘ankle’, ‘instability’ or ‘outcome’ and their synonyms (Appendix).

### Selection criteria

Articles were selected according to the following inclusion criteria: (1) patients were at least 18 years old at the time of surgery, (2) patients suffered from isolated lateral ankle instability for at least 6 months and were characterized by the subjective reporting of symptoms such as pain, swelling, instability and/or giving way, (3) patients were treated by some form of surgical stabilization, (4) described any of the following functional outcome measures at follow-up like pain, swelling, function, sport or quality of life.

Studies were excluded if they: (1) consisted of (systematic) reviews or case reports, (2) were not published in English, (3) only covered treatment of acute instability, (4) included medial instability, (5) only included conservative treatment, or (6) included patients with concomitant injuries, deformities or previous surgical treatment for ankle instability.

### Study selection

First all articles were screened by title and abstract for eligibility by two independent researchers. Next, the full-texts of the included articles were checked to determine whether they met the inclusion criteria. All articles of which full-texts were unavailable were excluded. Subsequently, all full-texts were read by two independent researchers and included or excluded based on the selection criteria. In case of disagreement, consensus on inclusion was reached during a meeting.

The final selection of included articles was scored according to the modified Coleman Scale for Methodology [35]. Each article was scored on study type, patient selection, diagnostics, treatment and assessment. The Coleman Score ranges from 0 to 90 referring to the methodologic quality, with a higher score representing better methodologic quality. Points were scored for number of included patients (0–10 points); mean follow-up (0–5); number of different procedures studied (0–10); type of study (0–15); diagnostic certainty (0–5); description of given treatment (0–5); outcome criteria (0–10); procedure used for assessing outcomes (0–15); description of subject selection process (0–15). The Modified Coleman Score (MCS) does not specifically include the rehabilitation process. In current studies, mostly the aftercare in terms of cast/bandage, etc. has been described, but details of the rehabilitation protocol have often not been reported. As our focus was on treatment and functional outcome and to avoid scoring bias due to underreporting of the rehabilitation protocol we therefore chose to use the MCS.

### Data extraction and statistical analysis

Two researchers reviewed all the included articles independently and extracted article characteristics, patient demographics, patient history, surgical treatment and questionnaires/scales used (including pre- and post-operative outcome).

To analyse baseline characteristics, the name of the main author, year of publication, study design, number of included patients and intervention were extracted.

To determine the best surgical procedure for treatment of CAI, outcome scores and outcomes (e.g. mean/median, SD/range) were extracted per procedure and article. In case reported outcomes were only shown as graphs, the mean/median and SD/range were estimated from the graphs. If studies included merely post-operative questionnaire scores, these questionnaires were only included in the qualitative analysis. Studies reporting both pre- and post-operative scores were pooled based on their mean scores and their mean score improvement. Using these means a weighted mean was calculated. Improvement per technique and superiority of a technique was evaluated using the independent *t* test. Questionnaires had to be used in at least two studies that assessed the same technique to make them eligible for pooling. If not, these articles were only used in the qualitative analysis. For pooling, Review Manager was used (RevMan [Computer program] version 5.3, Copenhagen: The Nordic Cochrane Centre, The Cochrane Collaboration, 2014) For statistical analysis SPSS was used (version 23.0, IBM Corp. Armonk, NY, USA). A *p* value of < 0.05 was considered statistically significant.

To assess heterogeneity between study population (number of patients, age, gender distribution and follow-up period), *I*^2^ was calculated [19]. In studies assessing the same technique using the same outcome scores, statistical pooling was performed. Pooling was only performed with patient-reported outcome measures (PROM) scores per technique.

## Results

### Study and patient characteristics

**Fig. 1:**
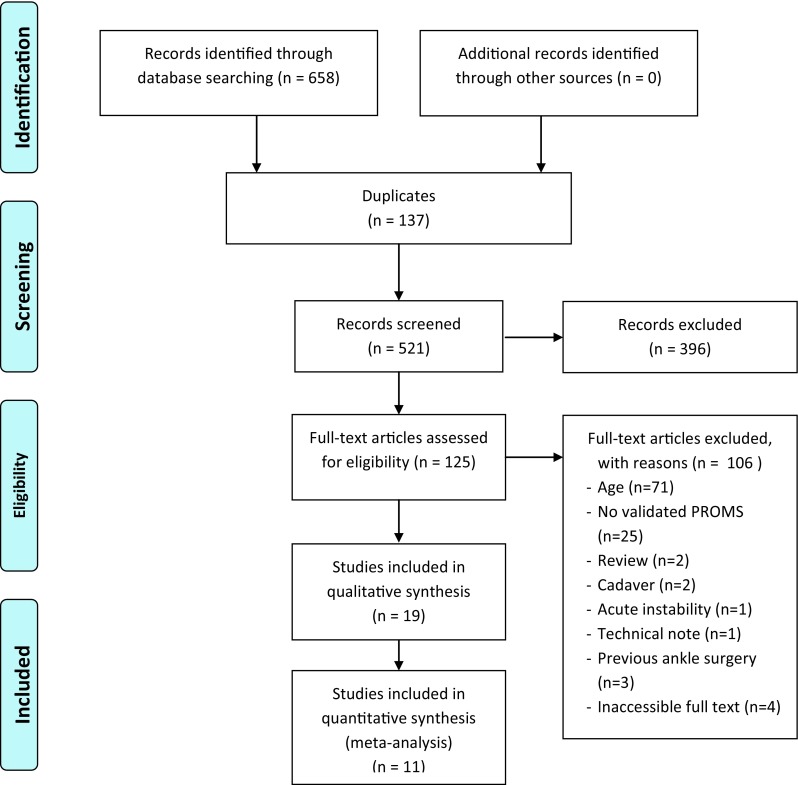
Flow chart included studies

The initial search provided 658 articles. After exclusion of irrelevant articles by screening the abstracts and subsequently reading the full-texts that remained, a total of 19 articles were included of which 11 were eligible for pooling of outcome data in the quantitative analysis (Fig. 1). Publication dates of included articles ranged from 2000 to 2015. The majority of the studies, 10 of 19, concerned retrospective cohort studies. Of five out of the 19 studies it was unknown whether the study design was prospective or retrospective. Articles that used any form of patient-reported outcome measure that was used by less than 3 of the included studies (per surgical procedure) were not included in the pooled results (Table 1).

**Table 1:** Patient characteristics

General information		Study design					Surgery			
References	Year of publication	Design	*N* (patients)	Gender (M:F)	Mean age (SD/range)	Mean follow-up (months; SD/range)	Stabilization technique	Surgery type	Additional procedures performed	Questionnaires used
Baray et al. [3]	2014	Retrospective	21	21:9	30.6 (± 12.4)	18 (± 8)	Hemi-Castaing procedure	TD	NR	Tegner; AOFAS; Karlsson
Bell et al. [5]	2006	NR	22	NR	20.7 (18–23)	26.3 (24.6–27.9)	Bröstrom	Arep	NR	SANE; FAOS, AOFAS
Caprio et al. [8]	2006	NR	11	8:3	NR	14.1 (NR)	Reconstruction with allogeneic semitendinosis graft	AR	NR	AOFAS; SF-12
Cho et al. [9]	2012	Prospective					Modified Bröstrom	Arep		
			20	12:8	33.9 (21–42)	28.4 (24–33)	Transosseous suture		10	Karlsson
			20	11:9	30.7 (15–44)	29.2 (24–34)	Suture anchor		9	Karlsson
de Vries et al. [11]	2008	Prospective	39	19:20	43.2 (± 11.1)	9 (NR)	Capsular shrinkage	CS	39	Karlsson; SF-36
Ellis et al. [15]	2011	Retrospective	11	4:7	48.9 (± 11.4)	42 (± 12.4)	Anatomic lateral ligament reconstruction with tendon allograft (n.s.)	AR	NR	FAOS; SF-36; Karlsson
Hu et al. [20]	2013	Prospective					Modified Bröstrom	Arep		
			40	32:8	34.8 (20–50)	34.2 (24–72)	Bone tunnel		21	AOFAS; Karlsson
			41	33:8	33.3 (19–52)	32.8 (24–64)	Suture anchor		26	AOFAS; Karlsson
Hua et al. [21]	2012	NR	35	*	29.2 (18–52)	37.9 (24–54)	Reconstruction with semitendinosis allograft	AR	NR	AOFAS; Karlsson
Hyer et al. [22]	2004	Retrospective	4	3:1	29 (20–32)	6 (NR)	Capsular shrinkage	CS	NR	Modified AOFAS
Ibrahim et al. [23]	2007	NR	14	21:2	25 (18–29)	NR	Reconstruction with gracilis tendon	AR	NR	AOFAS; VAS; Karlsson; OMAS
Kim et al. [24]	2015	Prospective	29	24:5	Med 24 (19–46)	21 (23–51)	Reconstruction with peroneus tendon	TD	26	Karlsson
Krips et al. [26]	2002	Retrospective	54	32:22	25 (± 4.2)	19.9 (± 3.6)	Karlsson technique	Arep	NR	Karlsson
			45	30:15	24 (± 4.2)	21.8 (± 4.6)	Evans tenodesis	TD	NR	Karlsson
Mabit et al. [30]	2010	Retrospective	310	164:146	28 (± 10)	156 (60–360)	Duquennoy; augmented repair; (hemi-) Castaing	CS	NR	Karlsson; Tegner
Maffulli et al. [32]	2013	Retrospective	38	NR	25.3 (18–41)	104.4 (± 32.5)	Bröstrom	Arep	NR	AOFAS; Kaikkonen Score
Ng and Das De [34]	2007	NR	20	20:0	23 (19–35)	12 (6–20)	Bröstrom–Evans–Gould	TD	NR	Kaikonen
Oloff et al. [36]	2000	Retrospective	10	7:9	34.5 (± 9.26)	9.6 (± 5.44)	Capsular shrinkage	CS	NR	AOFAS
Schepers et al. [39]	2011	Retrospective	20	12:8	33.9 (± 11.6)	109.6 (± 64.3)	Hemi-Castaing procedure	TD	NR	OMAS; KAFS; VAS; SF-36; Tegner
Ventura et al. [47]	2014	Retrospective	10	7:3	Med 29.4 (25–35)	16.3 (± 8.2)	Reconstruction with tendon allograft (n.s.)	TD	NR	AOFAS; Karlsson, Tegner activity level
Xu et al. [48]	2014	Retrospective	32	18:14	32.4 (± 2.4)	33.5 (± 6.7)	Reconstruction with semitendinosis autograft	AR	NR	AOFAS
			36	20:16	33.2 (± 3.2)	18.5 (± 6.7)	Reconstruction with tendon allograft (n.s.)	AR	NR	AOFAS

*NR* not reported, *Arep* anatomic repair, *AR* anatomic reconstruction, *TD* tenodesis, *CS* capsular shrinkage, *Med* median, *n.s.* not specified

* NR for the final study inclusion

A total of 882 patients were included in the studies described in the 19 articles with a mean of 44.4 patients per study (SD ± 59.3). Of the 882 included patients, 61% was male and 39% female. The mean age of included patients was 29.3 (SD ± 4.2), and a mean follow-up period of 76.0 months (SD ± 64.6) which varied greatly between articles (range 6–156 months). A total of 23 procedures were evaluated, including anatomic repair (*n* = 7), anatomic reconstruction (*n* = 6), tenodesis (*n* = 6) and capsular shrinkage (*n* = 4). Within the 23 different procedures, 5 different variations of tenodesis reconstruction were described, 4 of anatomic reconstruction, 4 of anatomic repair and 2 variations of performance of capsular shrinkage. In total, 6 studies performed additional procedures such as synovectomy, osteochondral debridement and microfracture, ossicle excision, loose body removal and bony spur resection. Only 5 articles (26%) mentioned the mean duration of symptoms, reporting a mean duration of symptoms of 31.6 months (SD ± 26.2) with a minimum duration of symptoms of 7 months and a maximum of 168 months.

### Critical appraisal and heterogeneity

**Fig. 2:**
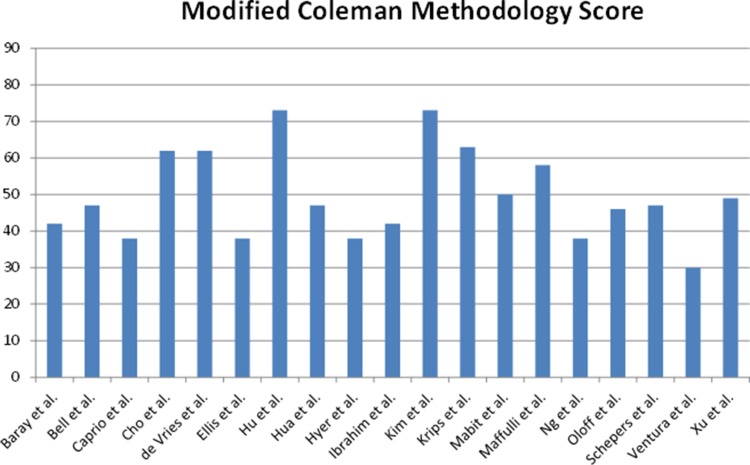
Quality assessment according to the Modified Coleman Methodology Score showing average quality of included articles with a large range in scores (30–73 on a scale of 0–90)

The included articles were scored using the Modified Coleman Methodology Scale with a maximum score of 90 points. The mean score was 49.6 points (SD ± 12.0) with scores ranging from 30 to 73 and no outliers, indicating that the included studies greatly vary in methodological quality (Fig. 2). The included articles mainly score low on the Modified Coleman Scale because of the low number of included patients, short follow-up periods, retrospective study designs and an insufficient or lack of description of the patient selection process.

**Fig. 3:**
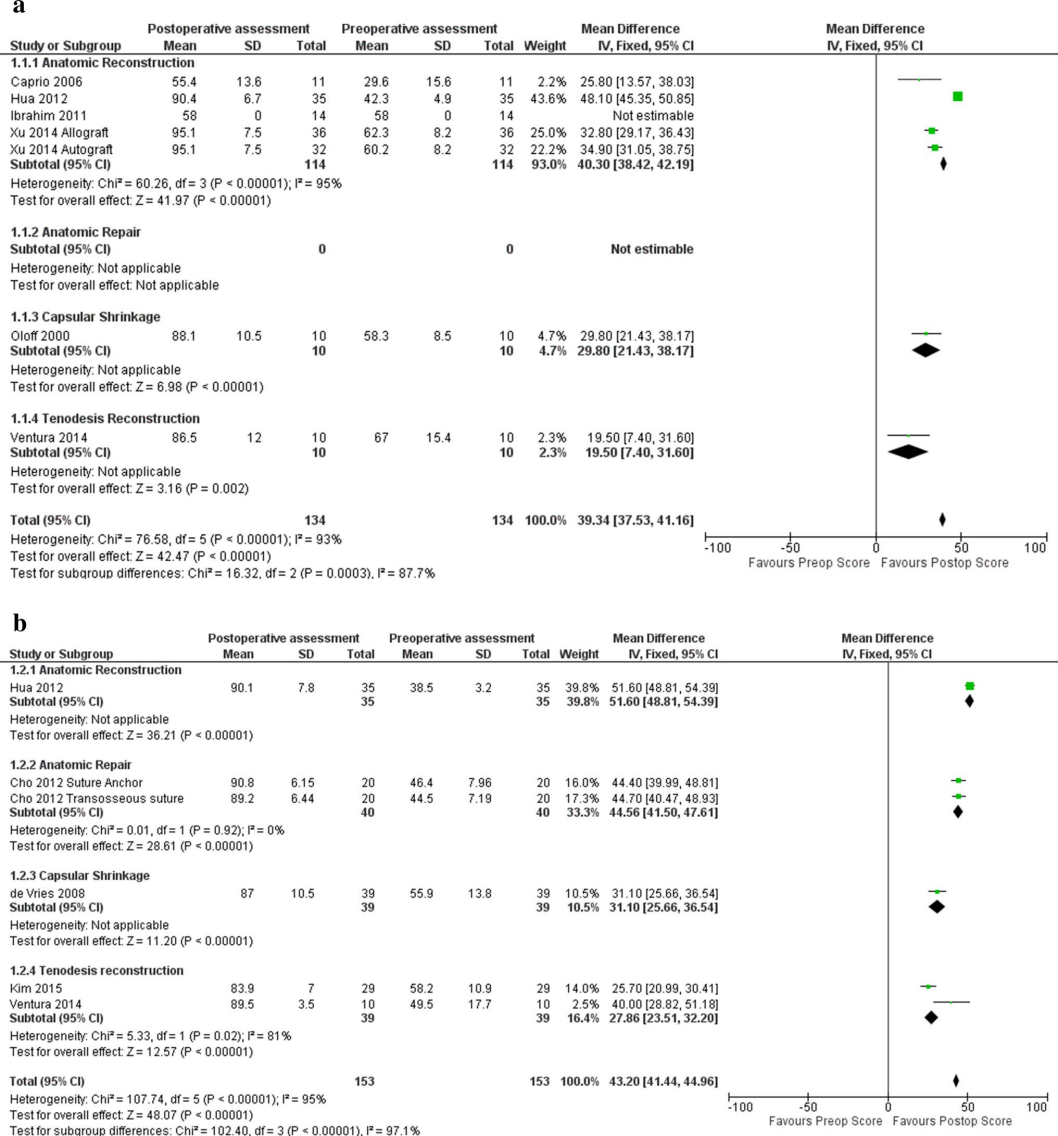
Forest plot pooled AOFAS (**a**) and Karlsson Scores (**b**). *AR* anatomic reconstruction, *Arep* anatomic repair, *CS* capsular shrinkage, *TD* tenodesis

The *I*^2^ on population heterogeneity (number of patients, mean age, male to female ratio, mean follow-up duration) was 19.9%, presenting no relevant heterogeneity in composition of the population. However, the inclusion of the outcome scores used for the analyses lead to 100% heterogeneity, reflecting the great number of different PROMs used. For the pooled data analyses, the heterogeneity varied from 93 to 95% (Fig. 3a, b).

### Patient-reported outcome measures

To assess surgical outcome a wide range of outcome scores were used, such as radiographic outcome, muscle function, ankle range of motion, but also joint laxity. In total, 11 different questionnaires were used to assess 23 procedures. In the 19 included studies, a total of 44 questionnaire-based outcome scores were available for analysis. The most commonly used questionnaires were the Karlsson Score (*n* = 13; 30.9%) and the AOFAS (*n* = 11; 26.2%). Only 25 (56.8%) out of 44 measurements were performed both pre- and post-operatively. Only 15 out of the 19 included studies reported whether the reported PROM score included a significant change.

The four studies that could not be pooled due to missing pre-operative scores reported overall good post-operative scores [5, 15, 26, 30]. The weighted mean of the post-operative Karlsson Score of these articles for anatomic repair was 83.7 (SD ± 10.4), for anatomic reconstruction 88.5 (SD ± 6.2) and for tenodesis 75.6 (SD ± 8.6). Other outcome scores were not reported frequently enough to calculate a weighted mean (Table 2).

**Table 2:** Outcome scores

Technique	References	Technique specifics	Questionnaire	Mean pre-op score (SD)	Mean post-op score (SD)
Anatomic reconstruction	Caprio et al. [8]		AOFAS	29.6 (15.6)	55.4 (13.6)
			SF-12	35.6 (9.14)	49.3 (8.7)
	Ellis et al. [15]		FAOS symptoms		80.4 (21.2)
			FAOS pain		85.2 (19.2)
			FAOS ADL		93.4 (10.5)
			FAOS sport		78.6 (NR)
			FAOS QoL		74.4 (NR)
			SF-36 physical		50.4 (8.2)
			SF-36 mental		45.0 (13.0)
			Karlsson		82.3 (19.9)
	Hua et al. [21]		AOFAS	42.3 (4.9)	90.4 (6.7)
			Karlsson	38.5 (3.2)	90.1 (7.8)
	Ibrahim et al. [23]		AOFAS	58.0 (30–70)	96.0 (80–100)
			Karlsson		94.7 (80–100)
			OMAS		87.5 (70–100)
			VAS	6.8 (3–10)	6.0 (NR)
	Xu et al. [48]	Autograft	AOFAS	62.3 (8.2)	95.1 (7.5)
		Allograft		60.2 (8.4)	94.2 (5.5)
Anatomic repair	Bell et al. [5]		AOFAS		89.4 (16.7)
			FAOS symptoms		93.6 (9.4)
			FAOS pain		93.9 (13.4)
			FAOS ADL		97.0 (9.2)
			FAOS sport		91.1 (18.0)
			FAOS QoL		87.2 (20.5)
			SANE		91.8 (10.2)
	Cho et al. [9]	Transosseous suture	Karlsson	44.5 (7.19)	89.2 (6.44)
		Suture anchor		46.4 (7.96)	90.8 (6.15)
	Hu et al. [20]	Bone tunnel	AOFAS	64.2 (53–73)	94.9 (77–100)
		Suture anchor		70.3 (57–87)	96.4 (85–100)
		Bone tunnel	Karlsson	57.0 (42–67)	97.8 (77–100)
		Suture anchor		59.9 (39–90)	97.4 (85–100)
	Krips et al. [26]	Anatomic repair	Karlsson		83.7 (10.4)
	Maffulli et al. [32]	Anatomic repair	AOFAS	51.0 (32–71)	90 (67–100)
			Kaikkonen	45.0 (30–70)	90.0 (65–100)
Capsular shrinkage	de Vries et al. [11]		Karlsson	55.9 (13.8)	87.0 (10.5)
			SF-36 physical	44.4 (8.9)	50.9 (9.1)
			SF-36 mental	51.3 (7.1)	52.0 (6.4)
			Tegner	2.9 (2.1)	4.5 (2.1)
			Good et al.	3.9 (0.3)	1.9 (0.8)
	Hyer and Vancourt [22]		Modified AOFAS	26.0 (10)	51.0 (8.8)
	Oloff et al. [36]	Capsular shrinkage	AOFAS	58.3 (8.5)	88.1 (10.5)
Tenodesis	Baray et al. [3]		AOFAS		88.1 (16.2)
			Karlsson		84.2 (23.8)
			Tegner	7.1 (3.2)	8.7 (3.6)
	Kim et al. [24]		Karlsson	58.2 (10.9)	83.9 (7.0)
	Krips et al. [26]		Karlsson		67.0 (15.8)
	Ng and Das De [34]	Tenodesis	Kaikkonen	39.8 (NR)	89.6 (NR)
	Schepers et al. [39]	Tenodesis	KAFS		79.4 (23.5)
			OMAS		83.7 (17.2)
			SF-36		109.2 (8.4)
			Tegner	4.15 (3.5)	4.3 (2.4)
			VAS		2.0 (2.4)
	Ventura et al. [47]	Tenodesis	AOFAS	67.0 (15.4)	86.5 (12.0)
			Karlsson	49.5 (17.7)	89.5 (3.5)
			Tegner	6.5 (NR)	7.0 (NR)
Combined techniques	Mabit et al. [30]	Tenodesis and anatomic repair	Karlsson		90.0 (19-100)

After pooling data per stabilization technique, all outcome scores showed post-operative improvement. Only four outcome scores were used often enough to assess whether there was significant improvement comparing the pre- and post-operative PROM scores (Table 3), i.e. the AOFAS, Karlsson, Kaikkonen and Tegner Score.

**Table 3:** Score improvement per technique

Technique	Questionnaire	*N* (patients)	References	Mean pre-op score (SD)	Mean post-op score (SD)	Mean score improvement (SD)	*p* value
Anatomic repair	AOFAS	119	Hu et al. [20] Maffuli et al. [32]	62.1 (8.0)	93.8 (2.7)	31.8 (5.3)	*p* < 0.0005
	Kaikkonen	38	Maffuli et al. [32]	45.0 (30–70)	90.0 (65–100)	45 (*)	*p* < 0.001
	Karlsson	121	Cho et al. [9] Hu et al. [20]	54.2 (6.3)	95.1 (3.6)	40.9 (2.9)	*p* < 0.0005
Anatomic reconstruction	AOFAS	128	Caprio et al. [8] Hua et al. [21] Ibrahim et al. [23] Xu et al. [48]	53.2 (10.6)	90.2 (10.9)	37.0 (6.8)	*p* < 0.0005
	Karlsson	35	Hua et al. [21]	38.5 (3.2)	90.1 (7.8)	51.6 (*)	*p* < 0.0005
Tenodesis	AOFAS	10	Ventura et al. [47]	67.0 (15.4)	86.5 (12)	19.5 (*)	*p* < 0.001
	Kaikkonen	20	Ng and Das De [34]	39.8 (NR)	89.6 (NR)	49.8 (*)	NR
	Karlsson	39	Kim et al. [24] Ventura et al. [47]	56.0 (3.8)	85.3 (2.5)	29.4 (6.3)	*p* < 0.0005
	Tegner	51	Baray et al. [3] Schepers et al. [39] Ventura et al. [47]	5.8 (1.4)	6.6 (2.0)	0.8 (0.7)	*p* < 0.0005

*NR* not reported, *SD* standard deviation

* Could not be calculated/unknown

Except for the mean post-operative AOFAS score of anatomic reconstruction compared to tenodesis (n.s.), all three techniques showed significant score changes comparing both the pre-operative post-operative outcome scores (*p* = 0.000–0.001). The highest post-operative scores were shown for anatomic repair as assessed by the AOFAS (93.8; SD ± 2.7) and Karlsson Score (95.1; SD ± 3.6). All outcome scores also showed significant improvement comparing pre- and post-operative scores (*p* < 0.001). Comparing pre- and post-operative questionnaire scores, all four studied techniques showed score improvement post-operative compared to the pre-operative situation (Fig. 3a, b). However, when comparing mean score improvement for anatomic repair, anatomic reconstruction and tenodesis, the greatest improvement was reported for anatomic reconstruction, followed by anatomic repair (*p* < 0.001–0.002) (Table 3; Fig. 3a, b).

## Discussion

The most important finding of the present study was better functional outcome after anatomic reconstruction and anatomic repair compared to tenodesis for operative treatment of chronic lateral ankle instability. Such a comparison could not be conducted earlier because of a lack of data. Due to the high number of different outcome scores used among studies, only anatomic reconstruction, anatomic repair and tenodesis reconstruction techniques could be quantitatively compared. Comparing patient-reported outcomes after surgical stabilization of the lateral ankle ligaments, all techniques showed relief of symptoms after surgical stabilization and improvement in PROM scores compared to pre-operative reports. Anatomic repair showed the highest post-operative scores. Despite overall improvement, tenodesis reconstruction showed the lowest scores.

Anatomic repair did not only provide higher post-operative outcome scores compared to anatomic reconstruction or tenodesis, it also showed higher pre-operative scores. This may be caused by selection bias. Even though anatomic repair is currently referred to as the ‘golden standard’, it can only be used when the tissue quality of the elongated ligament is sufficient for repair [6, 7, 10, 40]. In case of insufficient quality of the elongated ligaments, anatomic reconstruction is indicated. These cases might indicate a more severe instability on PROMs such as the Cumberland Ankle Instability Score. Higher initial scores of anatomic repair may reflect less severe instability. Techniques have changed over time, and so have surgical approaches and indication for treatment. Currently, tenodesis is mainly used as a salvage technique when other treatment choices are no longer viable options, compared to a few years ago when it was the primary treatment choice [4].

All techniques provide overall good results. For this reason, other factors may be taken into account when selecting the treatment. Patient preference may play a role in patient satisfaction [41]. The risk of complications and possible recurrence are other important factors to consider when choosing between treatment strategies. Anatomic repair may result in excellent post-operative outcomes, but its application is limited by the quality of remaining tissues [2, 37]. Tenodesis is often, as mentioned before, used as a salvage technique [4]. For this review, however, only studies were selected where patients had not yet undergone any form of surgical stabilization to filter out previous failed interventions and therefore avoid tenodesis being used for more severe indications.

While including outcome scores in the assessment, there was a high level of heterogeneity. This was caused by the number of different outcome scores used in the studies. When comparing the study populations a heterogeneity percentage of only 20% was calculated, meaning no important heterogeneity was present between the study populations. Hence, it was decided to pool the data with the aim to arrive at reliable conclusions, bearing in mind that the subgroups and high variety in used outcome scores affected study power.

The main limitation of this study is lack of power. There was a low number of studies per treatment type, a lack of pre- and post-operative assessment often without reporting of a SD or 95% CI, thus making data pooling impossible. Additionally, these studies used different outcome scores, again reducing power and increasing heterogeneity of the pooled data. Most studies were excluded based on participation of under-aged patients, performing multiple procedures at the same time or performing stabilization after failed initial surgery. To enable comparison of pre-operative assessments with post-operative assessments, minimizing bias due to unknown pre-operative scores, only the study outcomes that contained both outcome measures were pooled. This lead to a high number of studies being excluded from pooling data, again leading to a reduction of power [30]. An additional problem causing heterogeneity is patient selection for surgery as patients may suffer from mechanical and/or functional ankle instability. As functional instability is neuromuscular by nature, multiple factors are responsible for the feeling of giving way, possibly limiting the effect of surgery [25]. These studies were only included in the qualitative analysis. The quality of all included studies was low. Although the reported Coleman Scores were mainly around 55% of the scale, the population sizes of the individual studies were overall small and included too many outcome scores for the population size. This increased the chance of finding a coincidentally significant difference.

Despite these limitations and the different indications included in this meta-analysis, the strength of this review is the comparison of results per treatment modality. Comparability was enhanced by focusing only on first time surgery of CAI in adult patients. This may help treatment selection in case multiple treatment options are open.

In clinical practise, anatomic repair and anatomic reconstruction are preferred and should be the main treatment choice. Possibly with a slight preference towards anatomic repair in case the ligament remnants allow it, due to a minimal change in outcome with anatomic reconstruction. Additionally, if a repair fails, an anatomic reconstruction is still an option. Tenodesis reconstruction should be limited to salvage procedures only, when no other treatment option is open.

Implications for future research should include more high level studies such as randomized controlled trials on the outcome after different surgical stabilization procedures with a specific description of the population and use of minimum reporting standards advocated by the International Ankle Consortium [13, 17]. This may enhance comparability of both the indications and outcomes.

## Conclusion

In conclusion, anatomic reconstruction and anatomic repair provide better functional outcome based on PROM scores in patients treated by surgical stabilization for their ankle instability complaints, compared to tenodesis reconstruction and capsular shrinkage.

## Appendix: Search example PubMED

**Table:**

PubMed Search: (Reconstructive Surgical Procedures[Mesh] OR Surgical Procedures, Operative[Mesh] OR Tenodesis[Mesh] OR surger* [tiab] OR operation*[tiab] OR procedure*[tiab] OR tenodesis[tiab] OR reconstruction*[tiab] OR repair[tiab] OR Broström[tiab] OR Broström[tiab])
AND
(Ligaments[Mesh] OR ligament*[tiab])
AND
(Joint Instability[Mesh] OR anterior talofibular ligament instability*[tiab] OR ATFL instability*[tiab] OR anterior talofibular ligament laxity*[tiab] OR ATFL laxity*[tiab] OR calcaneofibular instability*[tiab] OR CFL instability*[tiab] OR calcaneofibular laxity*[tiab] OR CFL laxity*[tiab] OR instability*[tiab] OR laxity*[tiab])
AND
(Patient Outcome Assessment[Mesh] OR Patient Reported Outcome Measures[Mesh] OR Treatment Outcome[Mesh] OR outcome*[tiab] OR PROM*[tiab] OR patient reported outcome measure*[tiab]
